# General Signal Model for Multiple-Input Multiple-Output GMTI Radar

**DOI:** 10.3390/s18082576

**Published:** 2018-08-06

**Authors:** Fuyou Li, Feng He, Zhen Dong, Manqing Wu, Yongsheng Zhang

**Affiliations:** 1 School of Electronic Science, National University of Defense Technology, Changsha 410073, China; dongzhen@nudt.edu.cn (Z.D.); wumanqing@ustc.edu.cn (M.W.); zyongsheng6@gmail.com (Y.Z.); 2 China Academy of Electronics and Information Technology, China Electronics Technology Group Corporation, Changsha 410073, China

**Keywords:** MIMO GMTI radar, general signal model, fast-time waveforms, slow-time waveforms, range-compensation method

## Abstract

Multiple-input multiple-output (MIMO) ground moving target indication (GMTI) radar has been studied recently because of its excellent performance. In this paper, a general signal model is established for the MIMO GMTI radar with both fast-time and slow-time waveforms. The general signal model can be used to evaluate the performance of the MIMO GMTI radar with arbitrary waveforms such as the ideal orthogonal, code division multiple access (CDMA), frequency-division multiple access (FDMA), time division multiple access (TDMA), and Doppler division multiple access (DDMA) waveforms. We proposed a range-compensation method to eliminate the range-dependence of the FDMA waveforms. The simulation results indicate that the improved performance of FDMA waveforms is achieved utilizing the range-compensation method.

## 1. Introduction

Multiple-input multiple-output (MIMO) radar has been developed rapidly recently. Multiple waveforms are emitted by multiple transmitters, and thus more system degrees of freedom (DoFs) are achieved. Two classes of MIMO radar are included: statistical MIMO radar with widely separated antennas [1], and coherent MIMO radar with co-located antennas [2,3]. In this paper, coherent MIMO radar is taken into consideration. Coherent MIMO radar can achieve better performance compared with the single-input multiple-output (SIMO) radar: improved target detection performance and improved angle estimation accuracy [4]. In a MIMO radar, *M* signals are emitted by *M* transmitters, and the echoes are received by *N* receivers. For each receiver, echoes from different transmitters are separated by *M* bandpass filters or matched filters. Thus, MN channels are formed. Ground clutter is suppressed by a space time adaptive processing (STAP) technique in multi-channel ground moving target indication (GMTI) radar. Sharper clutter notches are achieved via the application of MIMO radar due to the increased DoFs and larger virtual array. Therefore, the minimum detectable velocity (MDV) will decrease in the MIMO GMTI radar [5].

Given the advantages of MIMO GMTI radar, the analysis and design of MIMO waveforms have recently been researched. In many publications, ideal orthogonal waveforms with good auto- and cross-correlations are assumed in the MIMO GMTI radar. Ideal orthogonal waveforms require the orthogonality at arbitrary time shifts instead of the orthogonality without shifts. In fact, it is difficult to design and implement ideal orthogonal waveforms in a fast amount of time [6]. The nonideal orthogonality of waveforms is the source of many problems in implementation of many MIMO GMTI radars. Based on the requirements of clutter suppression, to achieve good GMTI performance in MIMO radar, the coherent responses are required [7]. The orthogonality of most of the current waveforms is realized by space, time or frequency diversity. According to different time domains, the orthogonal waveforms can be grouped into fast-time and slow-time waveforms. The fast-time waveforms include code division multiple access (CDMA), frequency-division multiple access (FDMA); and the slow-time waveforms include time division multiple access (TDMA), and Doppler division multiple access (DDMA) [8]. Although the signal model for single waveform was established such as CDMA [9], FDMA [5], TDMA [10], and DDMA [11], the general signal model has not been well established. In order to analyze and compare the GMTI performance fairly in a general framework, a general signal model suitable for different waveforms is important and necessary in the MIMO GMTI radar. For the radar designers, a general signal model is more convenient for evaluating the performance of the MIMO waveforms and to choose applicable waveforms. The metric multiple-input single-output (MISO) cancellation ratio (MCR) is proposed for evaluating the performance of clutter suppression in [12], where MCR denotes an adaptive canceller and measures the clutter suppression of different MIMO waveforms. The GMTI performance is analyzed from the metric perspective but not from the signal model. The signal model for fast-time waveforms is established in [13]. In this model, the output signal-to-interference-plus-noise ratio (SINR) in STAP is influenced by waveform covariance matrix (WCM). However, the auto- and cross-correlation sidelobes may indirectly decrease the MIMO GMTI performance, and only the zero-lag WCM cannot satisfy the requirement of distributed clutter suppression. The signal model for slow-time waveforms is established in [7,14], and clutter covariance matrices and signal-to-noise ratio (SNR) loss performance for slow-time waveforms are discussed. This literature indicates that the ideal orthogonal fast-time waveforms don’t exist, and TDMA and DDMA can achieve good GMTI performance. The signal model for fast-time CDMA and slow-time waveforms are unified by an M×K space-time modulation matrix W [15], where *K* denotes the number of slow-time pulses in a coherent processing interval (CPI). However, the common FDMA waveform cannot be included in this model. A general signal model for both CDMA and FDMA is established to evaluate the MIMO GMTI performance [16]. This signal model analyzes the performance of the MIMO GMTI with different configurations. However, the slow-time waveforms aren’t included. In addition, this signal model adopts the model of a single scatterer. In practice, the responses of FDMA waveforms are dependent on the ranges of distribute clutter, thus the signal model is not applicable for FDMA in a distributed scene.

Now, there is not a general signal model, which can be used to analyze the advantages and disadvantages of different waveforms. In this paper, we focus on establishing a general signal model for the MIMO GMTI radar with arbitrary fast-time and slow-time waveforms, and evaluating the GMTI performance of these waveforms with this general model. The general signal model is started with transmitting signals, and the MIMO responses are expressed by the steering matrix and transmitting signals. The different waveforms correspond to different space-time modulation matrix and transmitting signals. Each class of waveforms is a special case in this general signal model. Based on this model, the performance of MIMO GMTI radar with different waveforms can be analyzed, and a relatively fair comparison can be made. In addition, the performance of the MIMO GMTI radar with different array geometries can be analyzed. According to this model, the essential reason why the FDMA cannot improve the GMTI performance is found. In distributed scene, the responses of FDMA are range-dependent. In other words, the responses of different range bins are not independently and identically distributed (IID), which is an important condition in STAP. After range compensation for FDMA MIMO GMTI radar, improved GMTI performance can be achieved.

## 2. Proposed General Signal Model

A general signal model for MIMO GMTI radar with different waveforms is established. The following notation is employed in this paper, scalars are denoted with italic typeface, and vectors (matrices) are denoted with bold lowercase (uppercase) typeface. Transposition, conjugation, and conjugate transposition are denoted by superscripts *T*, *, and *H*, respectively. MATLAB 2014a (MathWorks, USA) notations for vectors or matrices are employed, e.g., for two M×1 vectors a,b, C=[a,b] denotes an M×2 matrix, and c=[a;b] denotes an 2M×1 vector. ⊗, ⊙, diag(·), blkdiag(·) and E(·) denote Kronecker product, Hadamard product, diagonalization, block diagonalization, and expectation, respectively.

**Figure 1:**
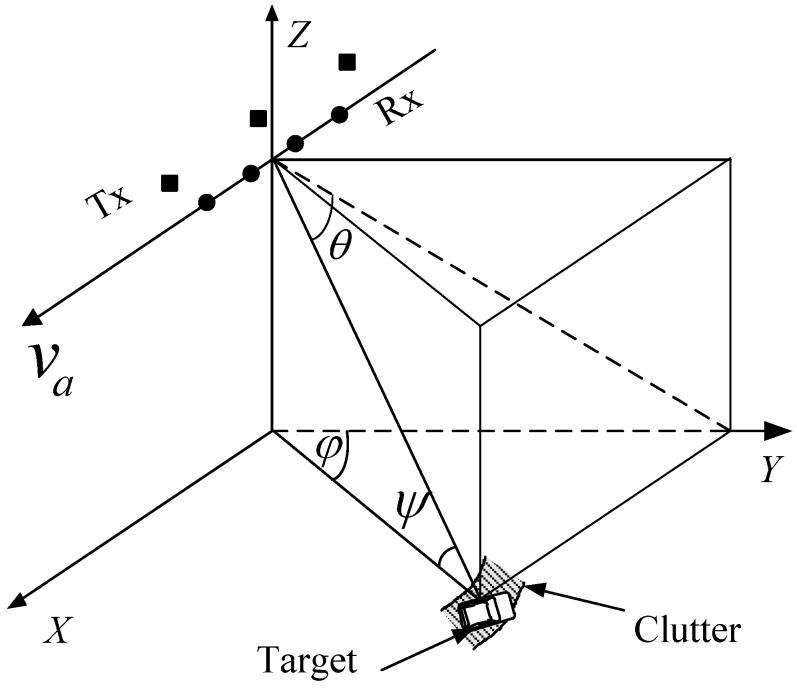
Illustration of the multiple-input multiple-output ground moving target indication radar.

Consider a sidelooking MIMO GMTI radar with *M* transmitters and *N* receivers, as depicted in Figure 1, and the radar system parameters are listed in Table 1. The nonideal factors as temporal fluctuation, channel mismatching are not considered. *M* transmitters emit *M* signals $S=[s_{T}^{\left(1\right)}\left(t\right),s_{T}^{\left(2\right)}\left(t\right),\cdots ,s_{T}^{\left(M\right)}\left(t\right)]$, where *m*-th transmitter emits the signal

(1) $$s_{T}^{\left(m\right)}\left(t\right)=s_{m}\left(t\right)\exp\left(j2\pi f_{m}t\right),$$

where $s_{m}\left(t\right)$, $f_{m}$ are the baseband signal and the carrier frequency. Assume the transmitting power is unity, $\int s_{T}^{\left(m\right)}\left(t\right)s_{T}^{(m)*}\left(t\right)dt=1$.

### 2.1. Range-Dependent Characteristic of Transmit Spatial Frequencies

Usually, the M×1 transmit steering vectors for a target at cone angle θ is denoted as

(2) $$a_{T\theta }=\exp\left\{j2\pi f_{m}d_{T}\sin\theta /c\right\},$$

where the cone angle θ satisfies sinθ=sinφcosψ, where φ, ψ are azimuth and grazing angles, respectively. This transmit spatial steering vector reflects the phase shifts introduced by transmitters locations [13]. However, for FDMA waveforms, the transmit spatial frequencies are variable with the ranges of targets or clutter patches [17,18]. Assume the carrier frequencies are $f_{m}=f_{1}+\left(m-1\right)\Delta \;f,m=1,\cdots ,M$. For the target with range *r* and cone angle θ, the phase shift of the 1st transmitter is

(3) $$\varphi_{1}=-2\pi f_{1}r/c,$$

the phase shift of the 2nd transmitter is

(4) $$\varphi_{2}=-2\pi f_{2}\left(r-d_{T,2}\sin\theta \right)/c,$$

where $d_{T,m}$, $d_{R,m}$ represent the *m*-th element of $d_{T}$, $d_{R}$, respectively. The distances of transmitters and receivers are relative, and we define $d_{T,1}=d_{R,1}=0$. Thus, the phase difference is represented as

(5) $$\varphi_{1,2}=\varphi_{2}-\varphi_{1}=-2\pi \Delta \;f\frac{r}{c}+2\pi f_{2}\frac{d_{T,2}\sin\theta }{c}.$$

Similarly, the phase shift of the *m*-th transmitter is

(6) $$\varphi_{m}=-2\pi f_{m}\left(r-d_{T,m}\sin\theta \right)/c.$$

Thus, the phase difference is represented as

(7) $$\varphi_{1,m}=\varphi_{m}-\varphi_{1}=-2\pi \left(m-1\right)\Delta \;f\frac{r}{c}+2\pi f_{m}\frac{d_{T,m}\sin\theta }{c}.$$

From the Equations (3)–(7), it follows that the phase difference is related to the ranges, so the transmit spatial frequencies are range-dependent. Therefore, the transmit steering vectors for the target with range *r* and cone angle θ is denoted by

(8) $$\begin{array}{cc} a_{T} & =a_{Tr}\left(r\right)⊙a_{T\theta }\left(\theta \right)\\ & ={\left[1,\exp\left\{-j2\pi \Delta \;f\frac{2r}{c}\right\},\cdots ,\exp\left\{-j2\pi \left(M-1\right)\Delta \;f\frac{2r}{c}\right\}\right]}^{T}\\ & ⊙{\left[1,\exp\left\{j2\pi f_{2}\frac{d_{T}\left(2\right)\sin\theta }{c}\right\},\cdots ,\exp\left\{j2\pi f_{M}\frac{d_{T}\left(M\right)\sin\theta }{c}\right\}\right]}^{T}. \end{array}$$

In Equation (8), 2r is used due to the double ranges in radar geometry. Based on Equation (8), the transmit steering vector and spatial frequencies are dependent on the ranges and cone angles. In the MIMO radar with the same carrier frequency (Δf=0), the relationship between transmit and receive spatial frequencies is linear [4]. However, in FDMA MIMO radar, the distribution of targets in transmit-receive spatial domains is arbitrary due to the range-dependent characteristic.

### 2.2. MIMO Responses

The responses of MIMO GMTI radar consist of amplitudes and phases. The phase shift is introduced by radar motion, target motion, locations of transmitters and receivers, and pulse time. The steering matrix represents the phase shift introduced by *M* transmitters, *N* receivers, and *K* pulses. In Ref. [19], the MIMO responses and the waveform matrix are related by a steering matrix, thus the signal model of the MIMO responses is simplified by a steering matrix denotation. For the *m*-th waveform, the N×1 receive steering vector is

(9) $$a_{R,m}=\exp\left\{j2\pi f_{m}d_{R}\sin\theta /c\right\}.$$

The K×1 temporal (Doppler) steering vector is

(10) $$a_{D,m}={\left[1,\cdots ,\exp\{j2\pi f_{m}2\left(v_{a}\sin\theta +v_{r}\right)\left(K-1\right)T_{r}/c\right]}^{T}.$$

It should be noted that antenna array misalignment with aircraft crab angle $\theta_{c}$ will change the Doppler frequencies into ${\bar{f}}_{d}=2v_{a}\sin\left(\varphi +\theta_{c}\right)\cos\psi /\lambda$. The impact on GMTI performance of the crab angle will be discussed next. Define an M×K space-time modulation matrix W denoting the amplitude and phase modulations for *M* transmitters and *K* slow-time pulses. The *m*-th row and *n*-th column element $w_{mn}$ of W denotes the amplitude and phase modulation of the *m*-th transmitter and *n*-th slow-time pulse (detailed discussions about W for different waveforms in Section 3). With the row partitioning of W, $W={\left[w_{1}^{T},w_{2}^{T},\cdots ,w_{M}^{T}\right]}^{T}$, where $w_{m}$ denotes the *m*-th row of W. Define M×MK matrix $\tilde{W}$ as the block diagonalization of W,

(11) $$\tilde{W}=\mathrm{blkdiag}\left(W\right)=\left[\begin{array}{ccc} w_{1} & & 0\\ & \ddots \\ 0 & & w_{M} \end{array}\right].$$

Define diagonal matrix T formed by $T=\mathrm{diag}(a_{T})$. Define MK×K matrix D formed by temporal steering vector,

(12) $$D={\left[\mathrm{diag}\left(a_{D,1}\right),\mathrm{diag}\left(a_{D,2}\right),\cdots ,\mathrm{diag}\left(a_{D,M}\right)\right]}^{T}.$$

The M×K phase shift matrix $A_{TD}$ caused by transmitters and slow-time pulses can be denoted by

(13) $$A_{TD}=T\tilde{W}D,$$

where T, $\tilde{W}$ and D represent phase shifts due to locations of transmitters, additional space-time modulation and slow-time pulses, respectively. Define M×N matrix R associated with receive steering vector,

(14) $$R={\left[a_{R,1},a_{R,2},\cdots ,a_{R,M}\right]}^{T}.$$

The phase shift matrix $A_{TD}$ are partitioned into rows $A_{TD}={\left[a_{TD,1}^{T},a_{TD,2}^{T},\cdots ,a_{TD,M}^{T}\right]}^{T}$, where $a_{TD,m}$ denotes the *m*-th row of $A_{TD}$, thus the M×NK steering matrix A for from *M* transmitters to *N* receivers for *K* pulses can be represented as

(15) $$A={\left[{\left(a_{TD,1}⊗a_{R,1}^{T}\right)}^{T},{\left(a_{TD,2}⊗a_{R,2}^{T}\right)}^{T},\cdots ,{\left(a_{TD,M}⊗a_{R,M}^{T}\right)}^{T}\right]}^{T}.$$

The steering matrix represents the phase shift characteristic of MIMO responses. For a scatterer with range *r*, cone angle θ, and radial velocity $v_{r}$ (for clutter patches, $v_{r}=0$), the MIMO responses of *N* receivers for *K* pulses are

(16) $$Y={\left(\Gamma ⊙A\right)}^{T}S\left(t-2r/c\right),$$

where $\Gamma \in C^{M\times NK}$ is the the complex amplitude of the scatterer in a transmit–receive–time snapshot:

(17) $$\Gamma =1_{M\times 1}\left(\alpha^{T}⊗1_{1\times N}\right),$$

where $\alpha ={\left[\alpha_{1},\cdots ,\alpha_{M}\right]}^{T}$ is the complex amplitude of the scatterer over a CPI. In the ideal environment, the amplitudes over a CPI are considered as a constant. However, the amplitudes of the moving targets and the clutter patches will vary with the slow-time samples. The variation can be modeled by the channel mismatch errors in Section 3.4.

From the signal model, the MIMO responses depend on the amplitude of the scatters α, the steering matrix A, and the waveforms matrix S. Both the fast-time and the slow-time waveforms can be included in the proposed signal model. The existing signal models can analyze only one class of waveforms, and can not compare the GMTI performance of the MIMO radar with different waveforms. The steering matrix for different waveforms is an important matrix to identify the different classes of waveforms.

### 2.3. Comparison with Other Signal Models

Although the signal model for a single waveform (CDMA, FDMA, etc.) exists, there is no signal model that can cover all the waveforms for the MIMO GMTI radar. In this paper, we establish a general signal model that is available for the MIMO GMTI radar with arbitrary waveforms. If only a waveform of the MIMO GMTI radar is given, we can analyze the GMTI performance using the proposed signal model. In addition, Equation (16) denotes the MIMO responses as the product of the steering matrix and the waveform matrix. It is very useful to optimize the waveforms under some constraints in the cognitive MIMO GMTI radar. The comparisons of the different signal models are listed in Table 2.

## 3. The Steering Matrix for Different Waveforms

**Figure 2:**
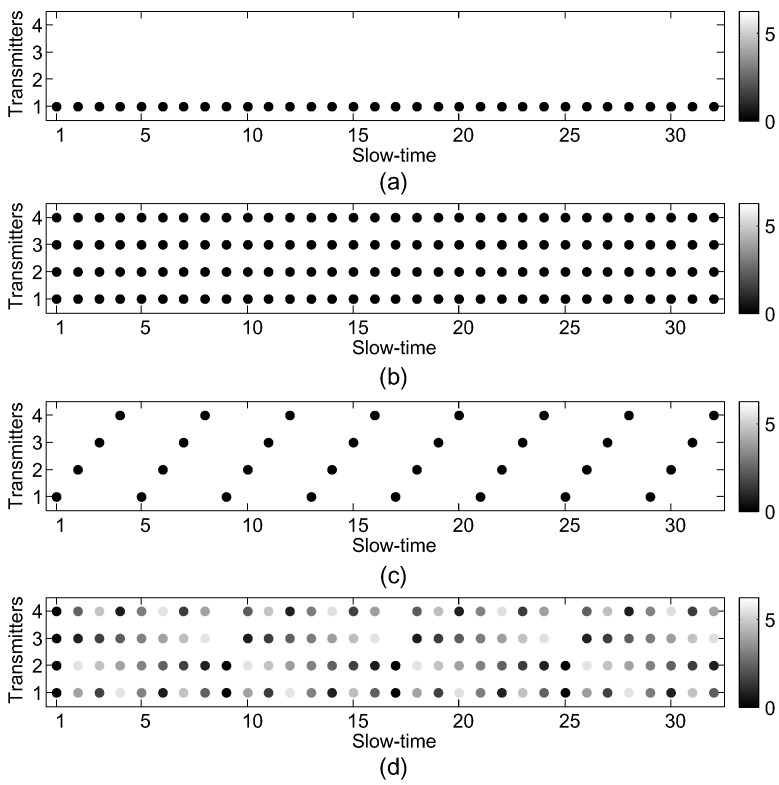
The radian phases of the space-time modulation matrix for (**a**) SIMO; (**b**) CDMA/FDMA; (**c**) TDMA; (**d**) DDMA.

In this section, the simple forms of different waveforms can be obtained. Each waveform is a special case of the proposed signal model. Based on the proposed general signal model for the MIMO GMTI radar, the MIMO responses are closely related to the the steering matrix, and the steering matrix is closely associated with the the space-time modulation matrix W. Different waveforms are corresponding to different forms of W. The two dimensions of W represent the transmit and slow-time aspects, respectively. Intuitively, the wrapped radian phases in [0,2π) of matrix W are shown in Figure 2 for different transmit schemes [15]. If only one row of W is all 1s, and the other elements are 0 as shown in Figure 2a, $W_{SIMO}=\left[0_{(M-1)\times K};1_{1\times K}\right]$, only one transmitter is activated in a CPI without slow-time modulation, then the MIMO degrades into SIMO. As Figure 2b, W is an all one matrix, $W_{CDMA/FDMA}=1_{M\times K}$. The waveforms such as CDMA, FDMA are emitted by all transmitters simultaneously in each pulse repetition interval (PRI). Due to the fast-time orthogonality without slow-time modulation, the echoes of these waveforms are separated in the fast-time domain. If only one transmitter is activated in each PRI, and the different transmitters are periodically toggled in a CPI [10], thus phases of W are shown in Figure 2c, and this waveform is called TDMA. The space-time modulation matrix of TDMA can be denoted as

(18) $$W_{TDMA}=\left[w_{ij}\right]=\left\{\begin{array}{cc} 1, & \mathrm{if}\;i+j=Mn+1,n\in N^{+},\\ 0, & \mathrm{others}. \end{array}\right.$$

As shown in Figure 2d, the slow-time phase modulation is applied in the MIMO GMTI radar. The same waveform is emitted by all the transmitters, and linear phases are appended in each transmitter. This waveform are called DDMA. The space-time modulation matrix of DDMA can be denoted as

(19) $$W_{DDMA}=\left[w_{mk}\right]=\exp\left\{-j2\pi \frac{f_{r}}{2M}\left(M+1-2m\right)\left(k-1\right)T_{r}\right\}.$$

Different Doppler frequencies for different transmitters are achieved by slow-time modulation, thus the echoes are separated by bandpass filters in the Doppler domain.

### 3.1. Slow-Time Waveforms

If each row of the space-time modulation matrix W is changed with the slow time, the orthogonality of MIMO waveforms are achieved in slow-time or Doppler domain. TDMA and DDMA both belong to slow-time waveforms.

For TDMA waveforms, only one transmitter is active in each PRI, and a periodic toggling of transmitters is adopted. Only one waveform with good autocorrelation is required. The TDMA waveforms are simple and easy to be implemented. In the system with the strict restriction of antenna, additional virtual phase centers can be generated at the cost of reduced transmit power, i.e., signal to noise ratio (SNR) will reduce in the same CPI as SIMO radar. In the signal model of the Section 2, Δf=0, and $W_{TDMA}$ is shown in Figure 2c. The steering matrix A in Equation (15) becomes

(20) $$A=\left[\mathrm{diag}\left(a_{T\theta }\right)W\mathrm{diag}\left(a_{D}\right)\right]⊗a_{R}^{T}.$$

Potential good GMTI performance can be achieved using TDMA waveforms due to the larger baseline. The effective pulse repetition interval (PRF) of TDMA waveforms $f_{r,TDMA}=f_{r}/M$ is low, especially in the system with large *M*. Compared to the SIMO system, the additional Doppler ambiguities arise due to the PRF reduction. If SNR can be counterbalanced by increasing the transmitting power, good GMTI performance can be achieved by TDMA waveforms [20].

Another slow-time waveform is called DDMA, which separates the echoes in the Doppler domain. Different phases are appended to each transmitter along slow time *k*. The element $w_{mk}$ of W at the *m*-th (1≤m≤M) row and *k*-th (1≤k≤K) column is denoted in Equation (19). In a DDMA system, the steering matrix is the same as Equation (20) except W. After range compression and echo separation, the steering vector is $a_{T}⊗a_{D}⊗a_{R}$, which has the same structure as the ideal orthogonal waveforms. This model is consistent with the slow-time signal model in [21]. Similar to the TDMA waveforms, sufficient PRF is required. Doppler ambiguities arise in the DDMA system with large *M* and/or large Doppler bandwidth of clutter and moving targets. High PRF will induce range ambiguities, which is not desirable for radar application. Some improved methods are proposed to mitigate the blind velocities [12,22].

### 3.2. Fast-Time Waveforms

If each row of the space-time modulation matrix W is unchanged with the slow time, the orthogonality of MIMO waveforms is achieved in fast-time by emitting *M* almost orthogonal waveforms. This class of waveforms is called fast-time waveforms. *M* signals are emitted by all the *M* transmitters simultaneously in every PRI. No slow-time modulation is employed, thus the elements of W are all 1 s.

First, the CDMA waveforms (Δf=0) are discussed. The steering matrix in Equation (15) becomes

(21) $$A=a_{T}\left(a_{D}^{T}⊗a_{R}^{T}\right).$$

Compared with Equation (20), the phase shifts without slow-time modulation are induced by locations of transmitters and receivers, and slow-time pulses. The echoes from different transmitters are separated by *M* matched filters where $S^{H}$ is used. Then, the filter output can be denoted as

(22) $$\tilde{Y}={\left(\Gamma ⊙A\right)}^{T}S\left(t-2r/c\right)S^{H}\left(t-2r/c\right)=\Gamma^{T}⊙\left[\left(a_{D}⊗a_{R}\right)\left(a_{T}^{T}C\left(0\right)\right)\right],$$

where C(τ) is the WCM:

(23) $$C\left(\tau \right)≜\int S\left(t+\tau \right)S^{H}\left(t\right)dt.$$

The signal model (22) is consistent with the model in [13]. The key factor influencing the MIMO GMTI performance with CDMA waveforms are the zero-lag WCM. For the distributed scene, the returned signals have a certain delay width. Assume that the strengths of the returned signals from different delays are equal, and the returned signals from different delays are uncorrelated. Thus, the filter output for the distributed scene can be denoted as

(24) $$\begin{array}{cc} \tilde{Y} & =\int \Gamma^{T}⊙\left[\left(a_{D}⊗a_{R}\right)\left(a_{T}^{T}C\left(\tau \right)\right)d\right]\tau \\ & =\Gamma^{T}⊙\left[\left(a_{D}⊗a_{R}\right){\left(C_{\Sigma }a_{T}\right)}^{T}\right], \end{array}$$

where $C_{\Sigma }=\int C\left(\tau \right)d\tau$. Compared with Equation (22), the accumulation of the WCM at all delays $C_{\Sigma }$ will affect the GMTI performance. From this, the sidelobes of the WCM will decrease the GMTI performance indirectly. Stacking all the columns of $\tilde{Y}$ into a vector, then

(25) $$y=\mathrm{vec}\left(\tilde{Y}\right)=\mathrm{vec}\left(\Gamma^{T}\right)⊙\left(C_{\Sigma }a_{T}\right)⊗a_{D}⊗a_{R}≜\gamma ⊙v_{s},$$

where the $v_{s}$ is the transmit–receive–time steering vector, and $\gamma =\mathrm{vec}\left(\Gamma^{T}\right)=1_{N\times 1}⊗\alpha ⊗1_{M\times 1}$. The clutter returns are approximated by the summation of discrete independent clutter patches; then, the echo of a range bin of interest is represented as

(26) $$c=\sum_{k=1}^{N_{c}}\gamma_{k}⊙v_{s,k}\left(\theta_{k},f_{D,k}\right),$$

where $\gamma_{k}$, $\theta_{k}$, $f_{D,k}$ are the complex amplitude, cone angle and Doppler of the *k*-th clutter patch, respectively. Since the echoes are IID, then the clutter covariance matrix is denoted as

(27) $$\begin{array}{cc} R_{c} & =E\left(cc^{H}\right)=\sum_{k=1}^{N_{c}}\rho_{k}v_{s,k}\left(\theta_{k},f_{D,k}\right)v_{s,k}^{H}\left(\theta_{k},f_{D,k}\right), \end{array}$$

where $\rho_{k}$ is the average power of the *k*-th clutter patch. Based on this signal model, MIMO STAP is applied to detect moving targets. From Equation (24) to (27), the WCM $C_{\Sigma }$ is the main factor influencing the performance of the MIMO GMTI radar with CDMA waveforms. It demonstrates that the large integrated sidelobe ratio (ISLR) of CDMA may also degrade the GMTI performance of MIMO radar [9,12]. Obviously, when $C_{\Sigma }=I$, the fast-time CDMA waveforms become ideal orthogonal waveforms.

For the FDMA waveforms, the signal model is denoted as Section 2. The echoes of FDMA can be separated by *M* bandpass filters in fast-time domain, thus the correlation of FDMA waveforms can be considered as zero, and the WCM $C_{\Sigma }=I$. The echoes after bandpass filters, range compression and column stacking can be denoted as

(28) $$y_{FDMA}=\gamma ⊙\left[a_{T,1}a_{D,1}⊗a_{R,1};a_{T,2}a_{D,2}⊗a_{R,2};\cdots ;a_{T,M}a_{D,M}⊗a_{R,M}\right]=\gamma ⊙v_{FDMA},$$

where $a_{T,m}$ are the *m*-th element of the transmit steering vector $a_{T}$, and $v_{FDMA}$ is the steering vector for FDMA MIMO radar. Similarly, the echo of a range bin is denoted as

(29) $$c_{FDMA}=\sum_{k=1}^{N_{c}}\gamma_{k}⊙v_{FDMA}\left(r,\theta_{k},f_{D,k}\right).$$

In [16], the performance of FDMA is almost identical to that of ideal orthogonal waveforms. However, some publications show that the FDMA waveforms cannot improve the performance of the MIMO GMTI radar. Ref. [7] shows that the decorrelation of clutter returns from different transmitting signals degrades the GMTI performance. Ref. [14] shows that the increased rank of clutter covariance matrices degrades the performance of the MDV. Limited MCR for FDMA is demonstrated in [12].

**Figure 3:**
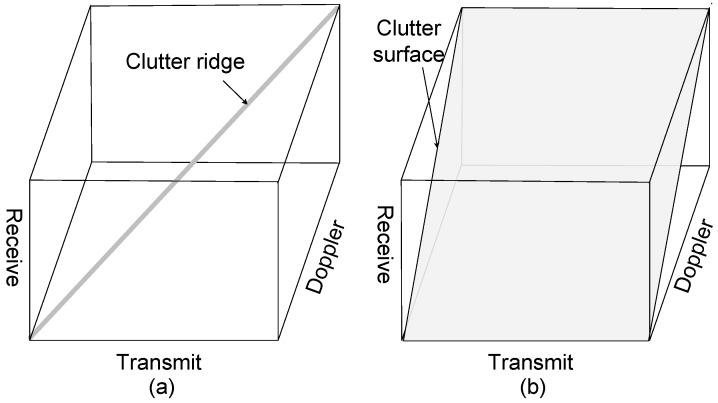
Clutter distribution in transmit–receive–Doppler space for (**a**) ideal MIMO orthogonal; (**b**) FDMA.

Comparing the proposed general model with the model in [16], the range-dependent characteristic of FDMA is not taken into consideration, and the echoes from different ranges are wrongly considered as IID in [16]. In practice, the echoes in Equation (29) are dependent on the ranges. According to the general signal model, it is found that the essential factor of performance degradation of FDMA is the range-dependent characteristic of the transmit spatial frequencies. The clutter distribution in transmit–receive–Doppler space is shown in Figure 3. The clutter of the MIMO GMTI radar with ideal orthogonal waveforms is coupled as a clutter ridge in transmit–receive–Doppler space; however, the clutter distribution of FDMA is a surface. The performance can be evaluated by the distance between the moving target and the clutter ridge or surface in transmit–receive–Doppler space. The distance of FDMA is closer than that of ideal orthogonal waveforms for a given target, thus the performance of FDMA is decreased. From another perspective, the entire transmit space of FDMA is filled with the clutter; hence, transmit diversity by FDMA doesn’t provide more efficient DoFs to suppress clutter. The echoes fail to meet the IID condition due to the range-dependent characteristic. Therefore, the rank of clutter increases, and the increase of transmit DoFs cannot improve the performance of MIMO GMTI radar.

Based on the transmit steering vector from Equation (3) to (8), the time delay 2r/c is the main reason that GMTI performance degrades. Utilizing the estimation method of the clutter covariance matrix in STAP, the clutter covariance matrix $R_{c,FDMA}$ can be denoted as

(30) $$R_{c,FDMA}=E\left[c_{FDMA}c_{FDMA}^{H}\right]=\frac{1}{N_{r}}\sum_{r}\left(\sum_{k=1}^{N_{c}}\gamma_{k}⊙v_{FDMA}\left(r,\theta_{k},f_{D,k}\right)\right){\left(\sum_{k=1}^{N_{c}}\gamma_{k}⊙v_{FDMA}\left(r,\theta_{k},f_{D,k}\right)\right)}^{H},$$

where $N_{r}$ is the number of the snapshots used to estimate the clutter covariance matrix. Comparing clutter covariance matrices of the FDMA with the CDMA waveforms, we can see that the clutter covariance matrix of the FDMA waveforms is associated with the ranges *r*, thus the rank of $R_{c,FDMA}$ in Equation (30) is larger than $R_{c}$ in Equation (27). Therefore, in order to eliminate the range-dependent characteristic, a range-compensation method for FDMA MIMO GMTI radar is proposed. According to the prior information of the range bins, a range-compensation vector is constructed as

(31) $$g\left(r\right)=a_{Tr}^{*}\left(r\right)={\left[1,\exp\left\{j2\pi \Delta \;f\frac{2r}{c}\right\},\cdots ,\exp\left\{j2\pi \left(M-1\right)\Delta \;f\frac{2r}{c}\right\}\right]}^{T}.$$

For the range bin with range *r*, the compensated echo data of FDMA can be denoted as

(32) $$\tilde{c}=\left[g\left(r\right)⊗1_{K}⊗1_{N}\right]⊙c_{FDMA}.$$

After range compensation, the range-dependent characteristic is removed, and the echoes from different range bins have the same distribution. Compared to SIMO radar, the system DoFs of MIMO radar with range-compensated FDMA waveforms increases due to the transmit diversity, and the clutter rank doesn’t increase, thus more dimensions can be used to suppress clutter in STAP and better GMTI performance is achieved.

### 3.3. Comparison of the MIMO GMTI Radar Waveforms

The goal of the signal model for MIMO GMTI radar is to establish the relationship between the transmit waveforms and the responses. The amplitudes and the phases are important to the MIMO responses. The steering matrix describes the phase shifts by the locations of transmitters and receivers, and the pulse time. The steering matrix is a bridge linking the transmit waveforms and the MIMO responses. The MIMO steering vector is directly related with the steering matrix. The MIMO steering vector of the ideal orthogonal waveforms is $a_{T}⊗a_{D}⊗a_{R}$. For CDMA waveforms, based on Equation (25), after matched filters, the steering vector is $\left(C_{\Sigma }a_{T}\right)⊗a_{D}⊗a_{R}$ for the distributed clutter. The accumulation of the waveform covariance matrix at all delays $C_{\Sigma }$ of CDMA waveforms is close to all-one matrix, so the elements of are approximately equal. Therefore, the transmit diversity of MIMO GMTI radar with CDMA waveforms disappears, thus CDMA waveforms are not suitable for distributed clutter. For FDMA waveforms, based on Equations (28) and (29), the steering vector is associated with the ranges of the clutter patches. For different range bins, the MIMO responses of the different transmit waveforms with different carrier frequencies are incoherent. Therefore, the responses of different range bins are not IID. FDMA waveforms can only achieve the same length of the baseline as the SIMO GMTI radar. The estimation of clutter covariance matrix of FDMA waveforms is close to the SIMO radar, so the MDV performance of FDMA waveforms is close to that of the SIMO radar. A range-compensation method to eliminate the range-dependence of the FDMA waveforms is implemented so that the echoes from different carrier frequencies are IID. The longer baseline can be achieved due to the different locations of transmitters via range-compensation. Thus, range- compensated FDMA can achieve better performance. For DDMA waveforms, after range compression and echo separation, the steering vector is $a_{T\theta }⊗a_{D}⊗a_{R}$, which is similar to the ideal orthogonal waveforms. The accuracy of the echo separation of DDMA is associated with the Doppler filters that can extract the echoes of different transmit waveforms relatively exactly. Therefore, DDMA is a choice for GMTI in distributed clutter. The comparison of different waveforms is shown in Table 3.

### 3.4. Other Real-World Factors Affecting MIMO GMTI Performance

In the practical environment, the idealized performance cannot be achieved usually. In this section, some real-world factors affecting the MIMO STAP performance such as channel mismatch, interference subspace leakage (ISL) from internal clutter motion (ICM) and range-walk, and antenna misalignment with crabbing are discussed. As seen in [23], the affection introduced by ISL and channel mismatch can be represented by a convenient mathematical model via covariance matrix tapering (CMT). If the interference covariance matrix is denoted as $R_{c}\in C^{MNK\times MNK}$, then the covariance including ISL and channel mismatch effects can be denoted as

(33) $${\tilde{R}}_{c}=R_{c}⊙T_{1}⊙T_{2}\cdots ⊙T_{k},$$

where $T_{1},T_{2},\cdots ,T_{k}\in C^{MNK\times MNK}$ are positive-(semi-) definite Hermitian matrices associated with uncorrelated with channel mismatch, ICM and range-walk). Next, the CMT structure will be considered in MIMO GMTI radar. A general expression denoting the CMT can be represented as

(34) $$T=T_{T}⊗T_{D}⊗T_{R},$$

where $T_{T},T_{D},T_{R}$ represent the transmit, time, and receive CMT, respectively.

Channel-mismatch errors include the gain errors often due to antenna gains and phase errors often caused by array geometries. The channel-mismatch errors usually remain stable over a CPI, thus the time CMT are an all-one matrix, $T_{D}=1_{K\times K}$. The transmit space mismatch taper is assumed as a vector vector random variable

(35) $$t_{T}={\left[\varepsilon_{1}e^{j\phi_{1}},\cdots ,\varepsilon_{M}e^{j\phi_{M}}\right]}^{T}.$$

The transmit space CMT can be denoted as

(36) $$T_{T}=E\left[t_{T}t_{T}^{H}\right].$$

To study the affection of amplitude and phase mismatch errors, a simple uncorrelated mismatch model is considered. The errors $\varepsilon_{i}$ and $\varepsilon_{i}$ subject to the uniform distributions $U(1-\Delta_{\varepsilon },1)$ and $U(-\Delta_{\phi },\Delta_{\phi })$, respectively. Then, the transmit spatial-only CMT can be calculated by

(37) $$T_{T}=\rho_{1}1+\rho_{2}I,$$

where $\rho_{1}={\left(1-\Delta_{\varepsilon }/2\right)}^{2}{\mathrm{sinc}}^{2}\left(\Delta_{\phi }/2\right)$, $\rho_{2}=1-\Delta_{\varepsilon }+\Delta_{\varepsilon }^{2}/3-\rho_{1}$ (details seen in [23]). Similarly, the receive spatial-only CMT can be calculated. The variation of the amplitudes over a CPI is equivalent to the amplitude error of the temporal steering vector, thus it can be modeled by applying a CMT to the temporal steering vector.

The other ISL as ICM can also cause an increasing rank of the Interference. Here, an empirical exponential model [24] is used to analyze the affection of ICM. The CMT caused by ICM can be denoted as $T=1_{M\times M}⊗T_{ICM}⊗T_{N\times N}$, where ${\left[T_{ICM}\right]}_{ij}=r_{c}\left(\left|i-j\right|T_{r}\right)$,

(38) $$r_{c}\left(\tau \right)=\frac{r}{r+1}+\frac{1}{r+1}\frac{{\left(b\lambda \right)}^{2}}{{\left(b\lambda \right)}^{2}+{\left(4\pi \tau \right)}^{2}},$$

where the parameter *b* is associated with wind conditions and has an empirical value in [24], and *r* is the solver of the following equation:

(39) $$10\log_{10}r=-15.5\log_{10}w-12.1\log_{10}f_{c}+63.2,$$

where $w,f_{c}$ are the wind speed in miles per hour (mph) and the carrier frequency in megahertz (MHz). Range walk due to the range migration of the clutter in a CPI will also cause ISL. The corresponding temporal CMT can be modeled as ${\left[T_{RW}\right]}_{ij}=\rho^{\left|i-j\right|}$, where ρ is the correlation between successive pulses, and can be approximated by the ratio of area overlap between successive pulses.

Antenna array misalignment with aircraft crabbing will cause altered clutter rank. The crab angle $\theta_{c}$ will introduce velocity misalignment. Doppler frequencies will changed into ${\bar{f}}_{d}=2v_{a}\sin\left(\varphi +\theta_{c}\right)\cos\psi /\lambda$. Thus, the relationship between normalized angle and Doppler is not strictly linear. Instead, the clutter locus forms an ellipse [25].

In the real–world environment, the clutter and targets are always nonhomogeneous. Power fluctuation, the interference targets and the discrete interference always exist in actual radar data. In STAP, the clutter covariance matrix is estimated by the training samples from different range bins. The clutter covariance matrix of STAP is estimated by

(40) $$R_{c}=E\left(cc^{H}\right)=\frac{1}{N_{r}}\sum_{k=1}^{N_{r}}c_{k}c_{k}^{H}.$$

In the real–world environment, the interference targets will affect the estimated clutter covariance matrix. Then, the estimated clutter covariance matrix will become

(41) $${\tilde{R}}_{c}=E\left(cc^{H}\right)=\frac{1}{N_{r}}\sum_{k=1}^{N_{r}}c_{k}c_{k}^{H}+\frac{1}{N_{r}}\sum_{j=1}^{J}{\left|\alpha_{j}\right|}^{2}t_{j}t_{j}^{H}=R_{c}+\frac{1}{N_{r}}\sum_{j=1}^{J}{\left|\alpha_{j}\right|}^{2}t_{j}t_{j}^{H},$$

where $t_{j}$ is the steering vector of the interference targets, and $c_{k}$ is the MIMO response of the stationary clutter patches that does not contain the nonideal factors as the interference targets and the discrete interference. In STAP, the interference contains the clutter, terminal noise, and interference moving targets. Thus, the interference moving targets will degrade the detection performance of the moving targets to be detected.

## 4. Simulation Results

In this section, the performance of the MIMO GMTI radar with fast- and slow-time waveforms are analyzed using the proposed general signal model. Consider a side-looking MIMO GMTI radar with four transmitters and four receivers. The platform height is H=10 km, the platform velocity is 100 m/s, the central grazing angle is $\psi =29^{\circ }$, and the central azimuth angle is $\varphi =0^{\circ }$. The direction of radar motion is parallel to the array antenna. The carrier frequency is 10 GHz. For the FDMA waveforms, the frequency step size is 111 MHz, for other waveforms, the step size is 0. The PRF is set as 6000 Hz. The separation spacings of transmitters and receivers are both 0.015 m (half-wavelength). In order to compare the system performance fairly, equivalent area search rates are assumed in our simulations. Since the area search rate is proportional to the beam size and inversely proportional to the number of pulses in a CPI, equivalent area search rates are tantamount to equal integrated SNRs. Assuming that the GMTI radar 1 has *q* times beam size as the GMTI radar 2, then we assume that the GMTI radar 2 utilizes *q* times as many pulses as the GMTI radar 1 so that the same clutter to noise ratios (CNR) is designed. In our simulations, we assume that the normalized power of the thermal noise is 1, the CNR is 30 dB, and the SNR is 30 dB.

### 4.1. Performance Evaluation of Different Waveforms

**Figure 4:**
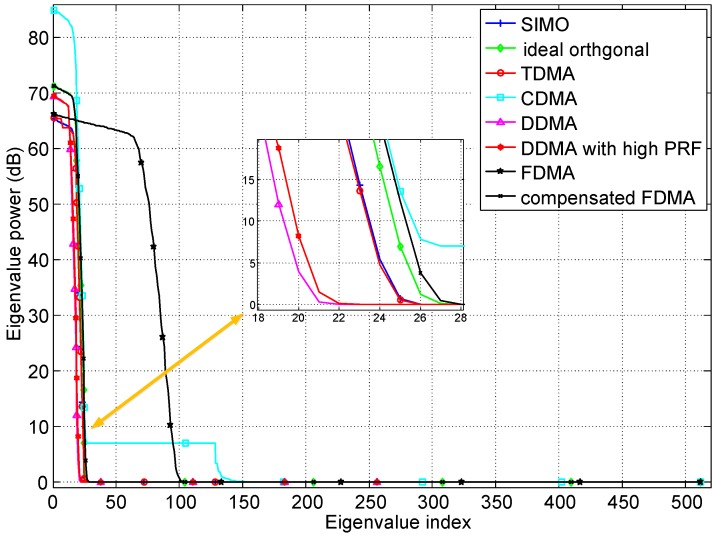
Eigenspectrums of MIMO GMTI radar with different waveforms.

Although the MIMO GMTI radar can provide more DoFs due to the transmit diversity to achieve better performance of clutter suppression, the clutter rank will also increase due to the waveform diversity. The clutter rank determined by clutter covariance matrix is associated with the waveforms, PRF and Doppler bandwidth, and the large clutter rank is adverse to suppress clutter [4]. Based on the proposed general signal model, the eigenspectrums of MIMO GMTI radar with different waveforms are plotted in Figure 4. The slow-time waveforms have smaller clutter rank compared to the other waveforms because the transmit diversity is achieved in Doppler domain, which decreases the system DoFs. The DDMA has smaller clutter rank due to smaller sub-PRF than the PRFs in SIMO and TDMA systems. It is important to note that the slow-time waveforms are closely related to the Doppler bandwidth decided by the beam width at a fixed carrier frequency. The sub-PRF of DDMA must be larger than the Doppler bandwidth. Because larger beam width or Doppler bandwidth can be utilized in systems with high PRF, thus the clutter rank of the DDMA with high PRF is larger than the usual DDMA waveforms. It can be predicted that the DDMA with high PRF will have smaller clutter rank compared to the usual DDMA system with the same beam width. The clutter rank of TDMA and SIMO is smaller than the MIMO waveforms, just because of smaller system DoFs. For the FDMA, the rank is about *M* times as large as the SIMO. Based on the analysis of the Section 2.1, the essential reason is the range-dependent characteristic of transmit spatial frequencies. The returned signals from different ranges are uncorrelated, so FDMA is not applicable to MIMO GMTI waveforms. The range-compensated FDMA eliminates the range-dependent characteristic, thus the returned signals from different ranges are IID. The clutter rank of compensated FDMA is almost the same as the ideal orthogonal waveform, and a little larger one is just caused by the multiple carrier frequencies of FDMA. Because the clutter rank has a smaller proportion of the system DoFs in the MIMO GMTI radar with ideal orthogonal or compensated FDMA waveforms, better GMTI performance can thus be achieved.

**Figure 5:**
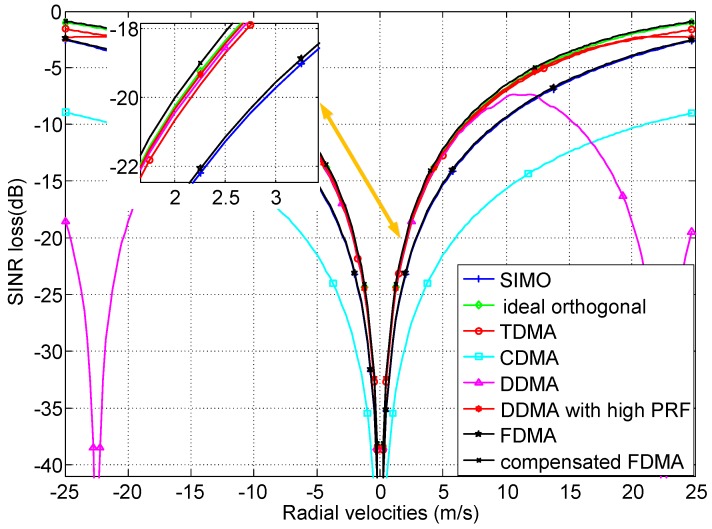
SINR loss of MIMO GMTI radar with different waveforms.

The SINR loss as a function of radial velocities of moving targets with azimuth angle $\varphi =0^{\circ }$ is shown in Figure 5. In these simulations, the range ambiguities are neglected for simplicity. CDMA waveforms have the worst performance because of the large ISLR, so they are inapplicable to be utilized in MIMO GMTI radar. Due to the Range-dependent characteristic of transmit spatial frequencies in FDMA MIMO GMTI radar, the system DoFs increase; unfortunately, the clutter rank increases the same multiple. The clutter rank occupies the same portion of the total system DoFs as SIMO radar, thus the SINR loss for FDMA is almost the same as SIMO GMTI radar. In the MIMO GMTI radar with TDMA, DDMA, DDMA with high PRF, compensated FDMA and ideal orthogonal waveforms, the clutter rank has a smaller portion of the total transmit–receive–time dimensions due to the introduced transmit diversity by MIMO techniques. Therefore, these classes of the MIMO GMTI radar with TDMA, DDMA, DDMA with high PRF and compensated FDMA have almost the same width of the clutter notches as the ideal orthogonal waveforms, which indicate that these classes of waveforms can achieve almost the same MDV performance. For the DDMA MIMO radar, although it has good MDV performance, the appearing blind velocities limit the performance of maximum detectable velocity. In addition, sufficient PRF is required in this system, otherwise the echoes from different transmitters cannot be separated [12,22]. The higher the PRF, the higher the maximum detectable velocity, as the DDMA waveforms with high PRF. However, the high PRF will introduce the range ambiguities, which will degrade the GMTI performance. Thus, the DDMA are well-suited to systems with high PRF, short to mid-range, and narrow Doppler bandwidth. For the TDMA waveforms, the additional phase centers are created to improve the GMTI performance by the cyclical toggling of the transmitters among pulses. If a higher signal power is emitted to counterbalance the SNR, the MDV performance can be improved by TDMA. Because the range-dependent characteristic is eliminated by range compensation, the compensated FDMA can achieve great improvement compared to the FDMA waveforms. The returned signals from different range bins are IID after range compensation, thus the clutter rank decrease. Therefore, the performance of SINR loss is almost not affected. Since the GMTI performance is sensitive to the carrier frequencies, and the carrier frequencies of FDMA are a little higher than the ideal orthogonal waveforms (caused by the frequency step Δf), the compensated FDMA is slightly better than the ideal orthogonal waveforms.

### 4.2. Performance Evaluation of Different Array Geometries

**Figure 6:**
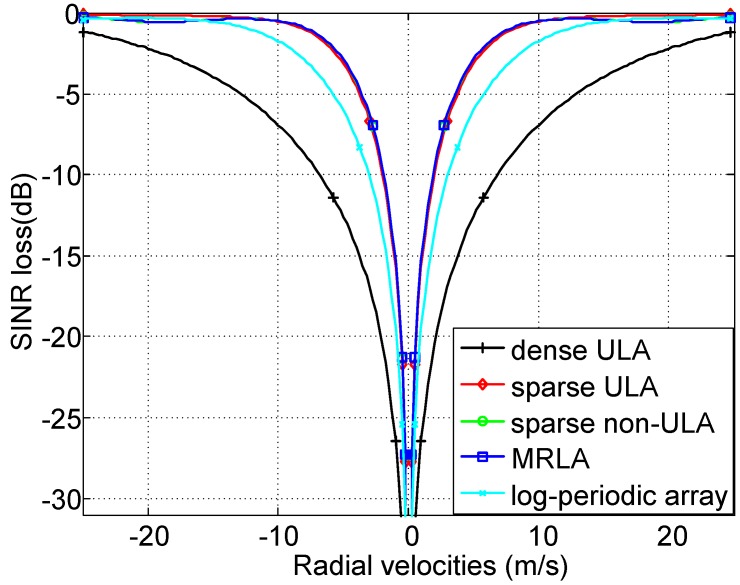
SINR loss of MIMO GMTI radar with different array geometries.

Based on the general signal model for the MIMO GMTI radar, the performance is influenced by array geometries including the transmitter locations $d_{T}$ and receiver locations $d_{R}$. MIMO virtual array is constructed by convolving $d_{T}$ and $d_{R}$ [4]. In this section, we assume the MIMO waveforms are ideal orthogonal, and analyze the performance of the MIMO GMTI radar with different array geometries. The other system parameters are the same as the previous except $d_{T}$ and $d_{R}$ shown in Table 4. The performance of different array geometries including dense uniform linear array (ULA) with spacing $d_{0}=\lambda /2$, sparse ULA with spacing $4d_{0}$, sparse non-ULA with locations ${\left[0,3d_{0},9d_{0},12d_{0}\right]}^{T}$, minimum redundancy linear array (MRLA) with locations ${\left[0,2d_{0},8d_{0},12d_{0}\right]}^{T}$, and log-periodic sparse array with locations ${\left[0,d_{0},3d_{0},7d_{0}\right]}^{T}$. The length of the virtual array of the MIMO GMTI radar with different array geometries is shown in Table 4. The SINR loss as a function of radial velocities of the moving target with azimuth angle $\varphi =0^{\circ }$ is shown in Figure 6. It demonstrates that the length of the virtual array is a key factor influencing the GMTI performance. The different array geometries with the same length of virtual array achieve almost the same performance, and the longer the length of the virtual array, the narrower the notches of SINR loss curves. In practice, the real array is limited by the cost, volume, and length, and so on. We should try our best to design the longest length of baseline among the transmit and receive antenna phase centers to improve the MIMO GMTI performance.

### 4.3. Performance Evaluation in Real-World Environments

In the real-world environment, some nonideal factors will influence the GMTI performance. To illustrate the impact of the nonideal factors affecting STAP performance on space-time clutter eigenspectrum and the SINR loss, consider the following factors: (1) Ideal, that is, no errors; (2) Channel-mismatch errors $\Delta_{\varepsilon }=0.01$, $\Delta_{\phi }=2^{\circ }$; (3) Channel-mismatch errors $\Delta_{\varepsilon }=0.02$, $\Delta_{\phi }=5^{\circ }$; (4) ISL from ICM with the wind speed 10 mph; and (5) ISL from ICM with the correlation ρ=0.9999. The other parameters are set as the previous.

**Figure 7:**
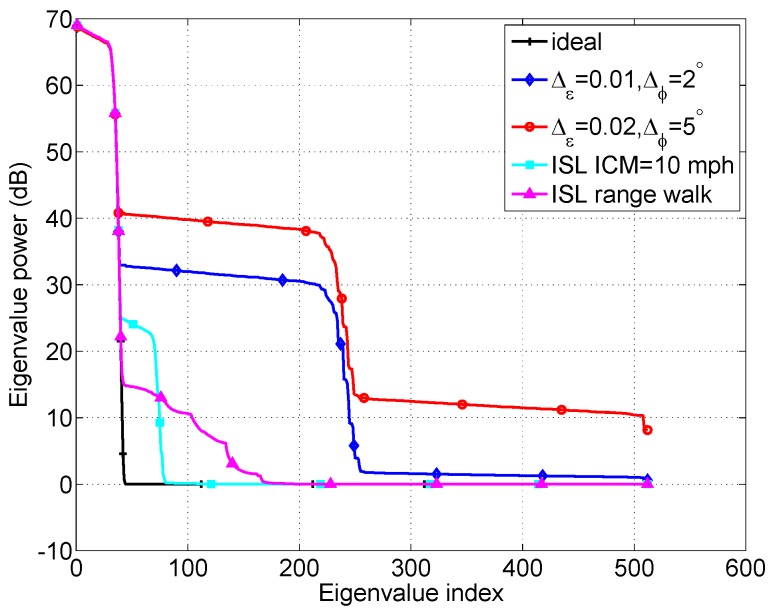
The impact of nonideal factors on the space-time clutter eigenspectrum.

**Figure 8:**
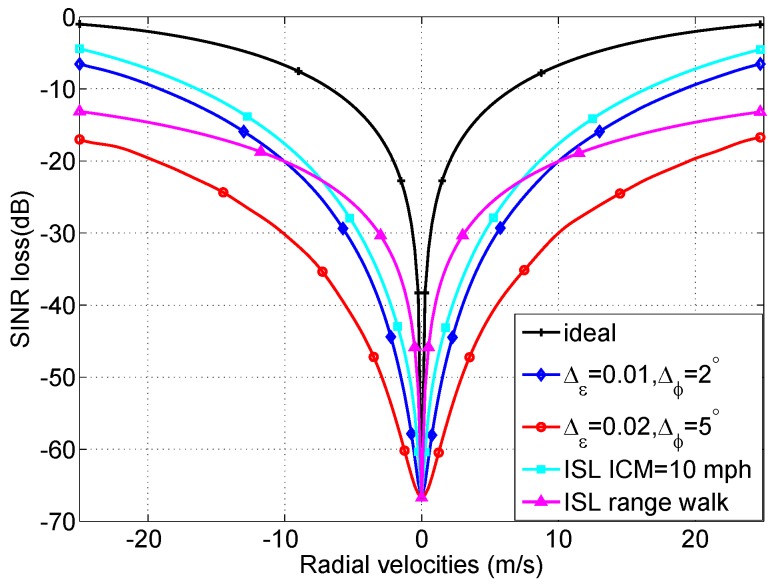
The impact of nonideal factors on the SINR loss.

**Figure 9:**
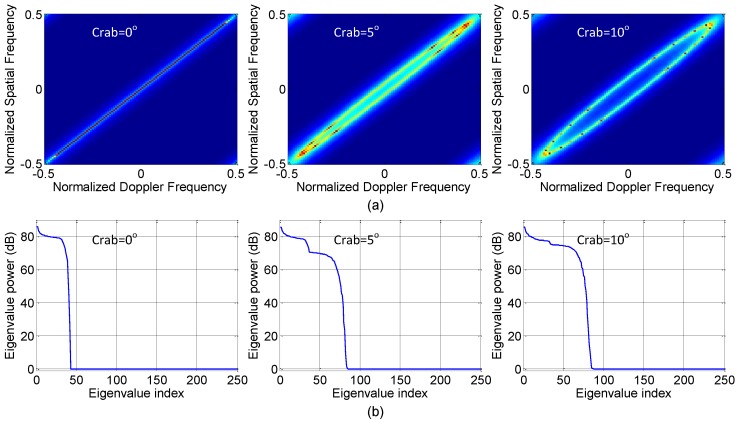
Effect of crabbing on (**a**) angle-Doppler image clutter; (**b**) eigenspectra, respectively.

The total clutter eigenvalues for the five cases are shown in Figure 7, and it shows that the clutter rank is increased due to the presence of the the nonideal factors affecting STAP performance, which will degrade the SINR performance. Figure 8 shows the SINR loss of the MIMO GMTI radar with these nonideal factors. The existence of the these nonideal factors will widen the mainbeam clutter notch, and thus degrades the detection performance of the slow targets, that is, the MDV is increased. Figure 9 displays angle-Doppler image and the corresponding clutter eigenspectrum for different values of crab angles. Note that the clutter ridges with crab angles become ellipses instead of a linear relationship of the normalized spatial frequencies and the Doppler frequencies. This is also deleterious to the detection of the moving targets.

**Figure 10:**
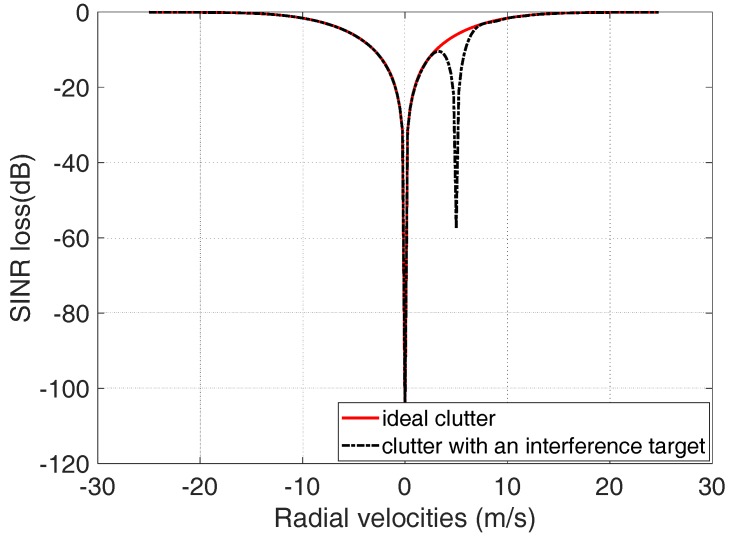
Comparison of the GMTI performance between the ideal clutter and the clutter with an interference target.

In the real-world environment, the interference moving targets will affect the performance of MIMO GMTI radar. Assume that a moving target with radial velocity 5 m/s is considered as an interference target in the scene. The comparison of the SINR loss with ideal clutter and with interference moving targets is shown as Figure 10. A notch of the SINR loss appears at the location with the radial velocity 5 m/s. The simulation results indicate that the interference target will degrade the GMTI performance of the moving targets with the radial velocity close to 5 m/s.

## 5. Conclusions

In this paper, we proposed a general signal model for the MIMO GMTI radar with arbitrary waveforms. The GMTI performance of the MIMO radar with fast- and slow-time waveforms can be conveniently analyzed through this general signal model. The performance curves of SINR loss for the MIMO GMTI radar with ideal orthogonal, TDMA, CDMA, DDMA, FDMA and range-compensated FDMA waveforms are plotted. Based on this general model, we can see that the performance of MIMO GMTI radar is decided by the estimation of clutter covariance matrix. The accurate estimation of clutter covariance matrix indicates good GMTI performance. A good waveform for MIMO GMTI radar requires good separation performance and the good coherence among the separated MIMO echoes. These two conditions are both necessary. The separated echoes of CDMA waveforms have good coherence, but its separation performance is bad; the separation performance of FDMA waveforms is good, but the coherence of separated echoes of CDMA waveforms is bad. Therefore, CDMA and FDMA are both not suitable for MIMO GMTI radar. A range-compensation method for the FDMA MIMO GMTI radar is proposed. The range-dependent characteristic is removed via range compensation, thus the MIMO GMTI performance is improved, and it is proved by the simulation results. Using this general signal model, the performance with different locations of transmitters and receivers associated with the length of the virtual array are also analyzed. Simulation results demonstrated that the key factor influencing the GMTI performance is the length of the virtual array instead of the number of equivalent phase centers. The real-world effects will degrade the performance of the MIMO GMTI radar, and the method solving the real-world factors will be our important work in the future.

## Figures and Tables

**Table 1:** Symbols for the multiple-input multiple-output ground moving target indication radar system parameters.

Parameter	Symbol
number of transmitters	*M*
number of receivers	*N*
number of pulses per CPI	*K*
radar carrier frequency	$f_{c}$
frequency step size for FDMA	Δf
speed of light	c
radar wavelength	$\lambda =c/f_{c}$
PRF	$f_{r}$
PRI	$T_{r}=1/f_{r}$
transmitters locations vector	$d_{T}$
receivers locations vector	$d_{R}$
platform velocity	$v_{a}$

**Table 2:** The comparison of the different signal models.

Signal Models	Descriptions and Limitations
Traditional ideal orthogonal model	This model assumed the MIMO radar transmits the ideal orthogonal waveforms in fast time, which is difficult to be designed and implemented. The nonideal orthogonal waveforms cannot be analyzed by this model.
Traditional CDMA model	Based on the traditional model, the factor influencing the GMTI performance is the waveform covariance matrix. Based on the proposed model, the exact factor should be the accumulation of the WCM at all the delays, and this signal model is unavailable to the FDMA waveforms.
Traditional FDMA model	The performance of the MIMO GMTI waveforms with stepped carrier frequencies are analyzed in this model. The FDMA model is not applicable to the CDMA waveforms, even if the stepped size of the carrier frequencies is set as 0 because the echoes from different waveforms with different carrier frequencies are usually considered to be orthogonal.
General signal model for CDMA and FDMA waveforms in [16]	A General signal model for both CDMA and FDMA MIMO GMTI radar is proposed in [16]. However, the range-dependent characteristic of the FDMA waveforms is not considered.
Traditional model for slow-time waveforms	Slow-time waveforms such as DDMA can be analyzed, but the fast-time waveforms cannot be analyzed by this slow-time model.
The unified model for fast-time CDMA and slow-time waveforms in [15]	The signal model for fast-time CDMA and slow-time waveforms are unified by a space-time modulation matrix W. However, the common FDMA waveform cannot be included in this model.
Proposed general signal model	The proposed general signal model is available for all the waveforms of the MIMO GMTI radar, such as FDMA, CDMA, TDMA, DDMA, and so on. In addition, the GMTI performance of different waveforms can be compared relatively fairly. In addition, the performance of the MIMO GMTI radar with different array geometries can be analyzed by this model.

**Table 3:** The comparison of MIMO GMTI radar waveforms.

Waveforms	Structure of the Steering Vector	Merits and Limitations
Ideal orthogonal waveforms	$a_{T}⊗a_{D}⊗a_{R}$	Best performance can be achieved, but it is difficult to be realized.
CDMA	$\left(C_{\Sigma }a_{T}\right)⊗a_{D}⊗a_{R}$	The performance is affected by $C_{\Sigma }$, so it is not suitable for distributed clutter. In cognitive radar, it can be used for the MIMO GMTI radar for the specific target.
DDMA	$a_{T}⊗a_{D}⊗a_{R}$	After range compression and echo separation, the structure of the steering vector is the same as the ideal orthogonal waveforms, so it is a choice of MIMO GMTI radar. Sufficient PRF freedom is required, so Doppler ambiguities will arise.
TDMA	$\mathrm{vec}([\mathrm{diag}\left(a_{T\theta }\right)W_{TDMA}$ $\mathrm{diag}\left(a_{D}\right)]⊗a_{R}^{T})$	TDMA waveforms are similar to DDMA waveforms. Sufficient PRF freedom is required. Compared to the other MIMO waveforms, the same transmit power required longer coherent processing time.
FDMA	$\left[a_{T,1}a_{D,1}⊗a_{R,1};a_{T,2}a_{D,2}⊗a_{R,2};\cdots ;a_{T,M}a_{D,M}⊗a_{R,M}\right]$	The steering vector is associated with the ranges of the clutter patches, so the echoes of different transmit waveforms with different carrier frequencies are not IID, which will degrade the GMTI performance. The blind velocities can be suppressed.
Range- compensated FDMA	$\left[a_{T\theta ,1}a_{D,1}⊗a_{R,1};a_{T\theta ,2}a_{D,2}⊗a_{R,2};\cdots ;a_{T\theta ,M}a_{D,M}⊗a_{R,M}\right]$	Compared to the traditional FDMA, the ranges are compensated, so the echoes from different ranges are IID. The transmit freedom is fully used. The GMTI performance is limited by the accuracy of the range compensation.

**Table 4:** Array geometries for MIMO GMTI radar.

Array Geometries	Location Vectors (m)	Length of Virtual Array (m)
dense ULA	$d_{T}=d_{R}={\left[0,0.015,0.03,0.045\right]}^{T}$	0.09
sparse ULA	$d_{T}=d_{R}={\left[0,0.06,0.12,0.18\right]}^{T}$	0.36
sparse non-ULA	$d_{T}=d_{R}={\left[0,0.045,0.135,0.18\right]}^{T}$	0.36
MRLA	$d_{T}=d_{R}={\left[0,0.03,0.12,0.18\right]}^{T}$	0.36
log-periodic sparse array	$d_{T}=d_{R}={\left[0,0.15,0.45,0.105\right]}^{T}$	0.21
